# Prevalence and correlates of anemia among children aged 6-23 months in Wolaita Zone, Southern Ethiopia

**DOI:** 10.1371/journal.pone.0206268

**Published:** 2019-03-08

**Authors:** Mihiretu Alemayehu, Mengistu Meskele, Bereket Alemayehu, Bereket Yakob

**Affiliations:** 1 School of Public Health, College of Health Sciences and Medicine, Wolaita Sodo University, Wolaita Sodo, Ethiopia; 2 Biomedical Science Division Wolaita Sodo University, College of Natural and Computational Sciences, Department of Biology, Wolaita Sodo, Ethiopia; 3 Department of Global Health and Population, Harvard T.H. Chan School of Public Health, Boston, MA, United States of America; BITS Pilani, INDIA

## Abstract

**Background:**

Anemia, the world’s most common micro-nutrient deficiency disorder, can affect a person at any time and at all stages of life. Though all members of the community may face the problem, children aged 6–23 months are particularly at higher risk. If left untreated, it adversely affects the health, cognitive development, school achievement, and work performance. However, little was investigated among young children in Sub-Saharan countries including Ethiopia. This research aimed to investigate its magnitude and correlates to address the gap and guide design of evidence based intervention.

**Methods:**

A community-based cross-sectional study was conducted from May -June 2016 in rural districts of Wolaita Zone. Multi-stage sampling technique was applied and 990 mother-child pairs were selected. Socio-demography, health and nutritional characteristics were collected by administering interview questionnaire to mothers/care-givers. Blood samples were taken to diagnose anemia by using HemoCue device, and the status was determined using cut-offs used for children aged 6–59 months. Hemoglobin concentration below 11.0 g/dl was considered anemic. Data were analyzed with Stata V14. Bivariate and multivariable logistic regressions were applied to identify candidates and predictor variables respectively. Statistical significance was determined at p-value < 0.05 at 95% confidence interval.

**Results:**

The mean hemoglobin level of children was 10.44±1.3g/dl, and 65.7% of them were anemic. Among anemic children, 0.4% were severely anemic (<7.0g/dl), while 28.1% and 37.2% were mildly (10.0–10.9g/dl) and moderately (7.0–9.9g/dl) anemic, respectively. In the multivariable analysis, having maternal age of 35 years and above (AOR = 1.96), being government employee (AOR = 0.29), being merchant (AOR = 0.43) and ‘other’ occupation (AOR = 3.17) were correlated with anemia in children in rural Wolaita. Similarly, receiving anti-helminthic drugs (AOR = 0.39), being female child (AOR = 1.76), consuming poor dietary diversity (AOR = 1.40), and having moderate household food insecurity (AOR = 1.72) were associated with anemia in rural Wolaita.

**Conclusion:**

A large majority of children in the rural Wolaita were anemic and the need for proven public health interventions such as food diversification, provision of anti-helminthic drugs and ensuring household food security is crucial. In addition, educating women on nutrition and diet diversification, as well as engaging them with alternative sources of income might be interventions in the study area.

## Introduction

Anemia is the world’s most common micro-nutrient deficiency disorder that affects more than 2 billion people globally [1]. It can affect a person at any time and at all stages of life. However, in most parts of the world, children aged 6–23 months are at particularly higher risk. It primarily affects infants and young children because of their higher iron requirements related to growth, and women of childbearing age due to menstrual loss and pregnancy [2].

If left untreated, anemia can adversely affect the health, cognitive development, school achievement, and work performance of individuals. Low oxygenation of brain tissues, a consequence of anemia, may lead to impaired cognitive function, growth and psychomotor development in children. Children under 5-year-old and pregnant women have greater susceptibility to anemia because of their increased iron requirements for rapid body growth and expansion of red blood cells [3]. During childhood period, anemia is strongly associated with poor health and physical development [4, 5], mild and moderate mental retardation [6], and poor motor development and its control [7]. Iron deficiency anemia leads to reduced academic achievement and work capacity which intern reduces the earning potential of individuals and hence damages national economic growth at large [5]. It also increases the risks of mortality and morbidity which come from other diseases [4, 8].

Childhood anemia is mostly caused by dietary iron deficiency, infectious and genetic diseases, and other nutrient deficiencies [9]. While more than half of the anemia burden in children is attributed to iron deficiency, only very small fraction is due to genetic causes [10]. In early childhood, bad feeding habits, especially during the weaning period as breast milk is replaced by foods that are poor in iron, vitamin B12 and folic acid, are main contributors for anemia [11].

In Ethiopia, evidence showed varying magnitudes of anemia ranging from 41% in Amhara region to 83% in Somali region [12]. Many studies showed that factors contributing to anemia in children varied from one geographical area to another [4, 6, 7, 10]. However, most of those studies were done using small sample size, conducted on urban dwellers, facility-based, and lacked representativeness. Besides, there was no study reported the problem in a wider context at district level in Woita Zone. Hence, the present study was conducted to fill the gap in a rural setting in Wolaita Zone with an aim of determining the magnitude of anemia and associated factors among children aged 6–23 months.

## Materials and methods

### Study design and study area

A community based cross-sectional study was conducted among children aged 6–23 months residing in rural districts of Wolaita Zone, Southern Ethiopia, from May to June 2016. Wolaita Zone is one of the 13 administrative zones of southern region (SNNPR) which has 12 rural and 3 urban districts. The Zone was inhabited by over 1.8 million people in 2016 [13]. Wolaita Sodo, the capital of the Zone, is located at 6° 49' N latitude and 39° 47' E longitude, at an altitude of about 1900 meters above sea level. It is located at 330 km south-west of Addis Ababa, Ethiopia. The zone is characterized by its dense population. The majority (88.3%) of its population reside in rural districts whose major livelihood is agriculture. The major food crops cultivated in the zone are maize, sweet potato, *enset* (false banana), *teff* (*Eragrostis tef*), haricot bean, taro, sorghum, Irish potato, yam and cassava [14].

### Sampling

Multi-stage sampling technique was applied to select mother-child paired study population. Children aged 6–23 months were the source population for the study. Initially, four districts were randomly selected from the 12 rural districts. Damot Gale, Boloso Bombe, Humbo and Sodo Zuria districts were selected as study districts. Then, three kebeles (the lowest administrative unit of Ethiopia consisting of nearly 5000 population) were randomly selected from each of the selected districts, making the total number of kebeles included in the study 12. Finally, the study participants were selected through systematic sampling technique from each of the selected kebeles by probability proportional to size i.e., allocating the sample size with regard to the respective kebeles’ population.

The total 993 sample size needed for this study was determined by the formula to estimate a single population proportion based on the following assumptions: 71.2% prevalence of anemia in Sub-Saharan Africa [15], 95% confidence interval, 5% margin of error, and 5% non-response rate and design effect of 3.

### Data collection tools and procedures

Data were collected through interviewer administered questionnaire prepared in English and translated into Amharic language). The questionnaire was developed by reviewing guidelines and related literatures [12, 15, 16, 17]. It consisted of demographic characteristics, household wealth indicators and anemia risk factors such as health service utilization, recent illnesses, and dietary practices of both mother and child.

#### Anemia diagnosis

Hemoglobin concentration was used to determine anemia status of the study participants by taking finger-prick blood sample. Hemoglobin level was analyzed onsite by using HemoCue device (HemoCueHb 301), and values were adjusted for altitude using the UNICEF/WHO guideline. Anemia status was determined using cut-offs used for children aged 6–59 months. Hemoglobin concentration below 11.0 g/dl was considered anemic, whereas, hemoglobin concentrations of 11.0 g/dl and above were considered normal. Severity of anemia was categorized based on the UNICEF, UNU, WHO guideline as follows: children were categorized as mildly, moderately and severely anemic if their blood hemoglobin concentrations are between 10.0–10.9 g/dl, 7.0–9.9 g/dl and < 7.0 g/dl, respectively. Maternal anemia (hemoglobin concentration below 12 g/dl) was diagnosed with the same procedure and device used for child anemia, followed by adjustment for pregnancy and altitude [16].

#### Dietary assessment

A 24-hour dietary recall method was used to assess the dietary practice. The Dietary Diversity Score of children was calculated by asking mothers/caregivers about the food items their children consumed in the past 24 hours preceding the survey. All food items consumed by the children in the last 24 hours preceding the survey were categorized into seven food groups such as (1) grains, roots, and tubers, (2) legumes and nuts, (3) milk and milk products, (4) flesh foods, (5) eggs, (6) vitamin-A rich fruits and vegetables, and (7) other fruits and vegetables. Finally, the food groups consumed by the child were added together to obtain dietary diversity score [17].

#### Food insecurity

Food insecurity was measured by HFIAS (Household Food Insecurity Access Scale) tool developed by FANTA (Food and Nutrition Technical Assistant) project. The tool has nine questions asking household’s about the three domains of food insecurity: feeling uncertainty of food supply, insufficient quality of food, and insufficient food intake and its physical consequences in the last month. The households participating in the study were categorized into four levels of food-security (food secure, mildly food insecure, moderately food insecure and severely food insecure) based on the guideline’s recommendation [18].

#### Wealth index

Household wealth index was constructed using household asset data through PCA (Principal Component Analysis) based on interview responses adopted from Ethiopian Demographic and Health Survey. The presence or absence of each household items such as plow oxen, table, animal-drawn cart, chair, etc. were asked and their responses were coded as ‘0’ for No and ‘1’ for Yes. Finally, the common factor score for each household was produced for grouping households as lower, middle and higher wealth quantile households [19].

#### Under-nutrition

Chronic energy deficiency (malnutrition) was assessed using WHO guideline. The WHO Anthro 2005 software was used to calculate *Z*-score. MUACZ cut-off- point of negative two (−2) was used to define under-nutrition [20].

### Data management and analysis

Data were entered into Epi-Data software version 1.4.4.0 and analyzed with Stata software version 14 (College Station, Texas). Proportions, means and standard deviations (SD) were used to describe the study population by independent variables and anemia. Bivariate logistic regression was done using thirty independent variables to identify the candidate variables (p-value < 0.25) for multivariable regression. Finally, predictors of anemia were determined using multivariable logistic regression model among the selected eleven candidate variables. Multi co-linearity, interaction and mediation among independent variables were checked based on the assumptions such as tolerance, variance inflation factors (VIF), correlation coefficient of interaction, and others to assure the fitness of logistic regression model. The independent variables used for checking multi co-linearity, interaction and mediation were; age of child, age of mother, religion, occupation, family size, meal frequency of mother, meal frequency of child, initiation of complementary feeding, receiving anti-helminthic drug, sex of child, dietary diversity score of child (DDS) and household food insecurity access scale (HFIAS). The statistical significance was determined at p-value < 0.05 at 95% confidence interval.

### Quality control

Interviewers and laboratory technicians were trained for two days prior to data collection. A pilot study was done among 50 children who were selected from areas outside the actual study area. The data collection was regularly supervised by trained supervisors and the investigators.

### Ethical approval and consent to participate

The actual data collection was started after Wolaita Sodo University College of Health Sciences and Medicine approved the study. Local administrative bodies were also communicated about the study and permission was obtained ahead of the study. Finally, informed written consent was obtained from the mothers/caregivers. Children diagnosed with anemia were counseled, and those with hemoglobin concentration below 9.0 g/dl were referred to health facility for further treatment and follow up.

## Results

### Socio-demography

A total of 990 children were included in the survey making the response rate 99.7%. The mean age of children was 14.96 months with a standard deviation of 5.37 months. About 434 (43.8%) of the children were females; 293 (29.6%) were born with short birth interval (less than 2 years interval); 191 (19.3%) were born from young mothers (mothers aged 15–24 years); and 60 (6.1%) were raised by single parents (Table 1).

**Table 1:** Socio-demographic characteristics of children aged 6–23 months residing in rural districts of Wolaita Zone, 2016 (N = 990).

Variable	Category	Number	Percent
Child age (in months)	6–11	336	33.9
	12–17	289	29.2
	18–23	365	36.9
Sex of child	Male	556	56.2
	Female	434	43.8
Birth interval	First born child	221	22.3
	below 24 month	293	29.6
	25–48 month	330	33.3
	above 48	146	14.8
Age of mother (in years)	15–24	191	19.3
	25–34	685	69.2
	35 and above	114	11.5
Marital status of mother	In a union	930	93.9
	Not in a union	60	6.1
Ethnicity of mother	Wolaita	963	97.3
	Others	27	2.7
Religion of mother	Protestant	857	86.6
	Orthodox	121	12.2
	Others^1^	12	1.2
Occupation of mother	Housewife	881	89.0
	Government employee	36	3.6
	Merchant	33	3.3
	Others^2^	40	4.0
Occupation of father	Farmer	624	63.0
	Government employee	111	11.2
	Merchant	175	17.7
	Others^2^	80	8.1
Home distance from health facility (foot walk)	Below 30 min	423	42.7
	30–45 min	443	44.8
	Above 45 min	124	12.5
Family size	Below 5	307	31.0
	5–7	451	45.6
	Above 7	232	23.4

Others^1^– Catholic, Muslim, Adventist

Others^2^ –Daily laborers, potters, housemaid

### Health and nutrition characteristics of children

The majority 922 (93.1%) of children were fully immunized and only 109 (11%) of them were given anti-helminthic drugs to treat intestinal parasites. A total of 409 (41.3%) children had at least one of the following symptoms within the past two weeks preceding the survey: cough, difficulty of breathing, fever, diarrhea (with/without blood), or visible parasite on stool. Based on the Mid-Upper Arm Circumference (MUAC) measurement, 688 (69.5%) of the children were well-nourished, whereas the rest 302 (30.5%) had chronic energy deficiency.

The children had mean dietary diversity score of 3.4 with 1.5 standard deviation, and only 422 (42.6%) of them consumed more than half of the recommended seven food groups. Furthermore, more than one-third (36.3%) drank either tea or coffee in their usual meal (that contained tannins which reduce iron absorption). Nearly one out of ten children stopped breastfeeding at least one week before the survey (Table 2).

**Table 2:** Health and nutrition characteristics of children in rural districts of Wolaita Zone, 2016 (N = 990).

Variable	Response Category	Number	Percent
Fully vaccinated	No	68	6.9
	Yes	922	93.1
Received anti-helminthes drug	No	881	89.0
	Yes	109	11.0
Sick in the past two weeks	No	581	58.7
	Yes	409	41.3
Ever caught malaria	No	642	64.8
	Yes	348	35.2
Usually drinks tea or coffee	No	631	63.7
	Yes	359	36.3
Bottle-feed	No	884	89.3
	Yes	106	10.7
Breast-feeding(currently)	No	110	11.1
	Yes	880	88.9
Complementary feeding frequency	1–3	507	51.2
(Within 24 hours)	4–5	440	44.4
	6–8	43	4.3
Breastfeeding frequency	1–6	194	19.6
	7–8	343	34.6
	Above 8	453	45.8
Initiation of complementary feeding	Before 6 month	90	9.1
	At 6 month	614	62.0
	After 6 month	286	28.9
Dietary diversity score	Below 4	568	57.4
	4 and above	422	42.6
Nutritional status (MUACZ)	≤-3	80	8.1
	Between -2 and -3	222	22.4
	Normal	688	69.5

Based on the household food insecurity access scale measurement (HFIAS), 628 (63.4%) of the children were born and lived in food-secure households; whereas the rest were born and lived in a household suffering from food insecurity. Four hundred and four (40.8%) mothers fully attended the four WHO recommended ANC visits, while only 86 (8.7%) of them had no ANC visit. The prevalence of maternal anemia was 13.4%, and three-fourth (734) of them usually consumed fewer than four meals per day (Table 3).

**Table 3:** Maternal health and nutrition in rural districts of Wolaita Zone, 2016 (N = 990).

Variable	Category	Number	Percent
Household food insecurity	Food secure	628	63.4
	Mildly food insecure	78	7.9
	Moderately food insecure	208	21.0
	Severely food insecure	76	7.7
Number of pregnancies	1–2	397	40.1
	3–4	298	30.1
	Above 4	295	29.8
Antenatal care follow-up	No visit	86	8.7
	1–3 visit	500	50.5
	4 and above	404	40.8
Place of delivery	Home	446	45.1
	Health facility	544	54.9
Maternal anemia	Yes	133	13.4
	No	857	86.6
Frequency of meals/day	Less than or equal to 3	734	74.1
	Above 3	256	25.9

### Magnitude and severity of anemia

**Fig 1:**
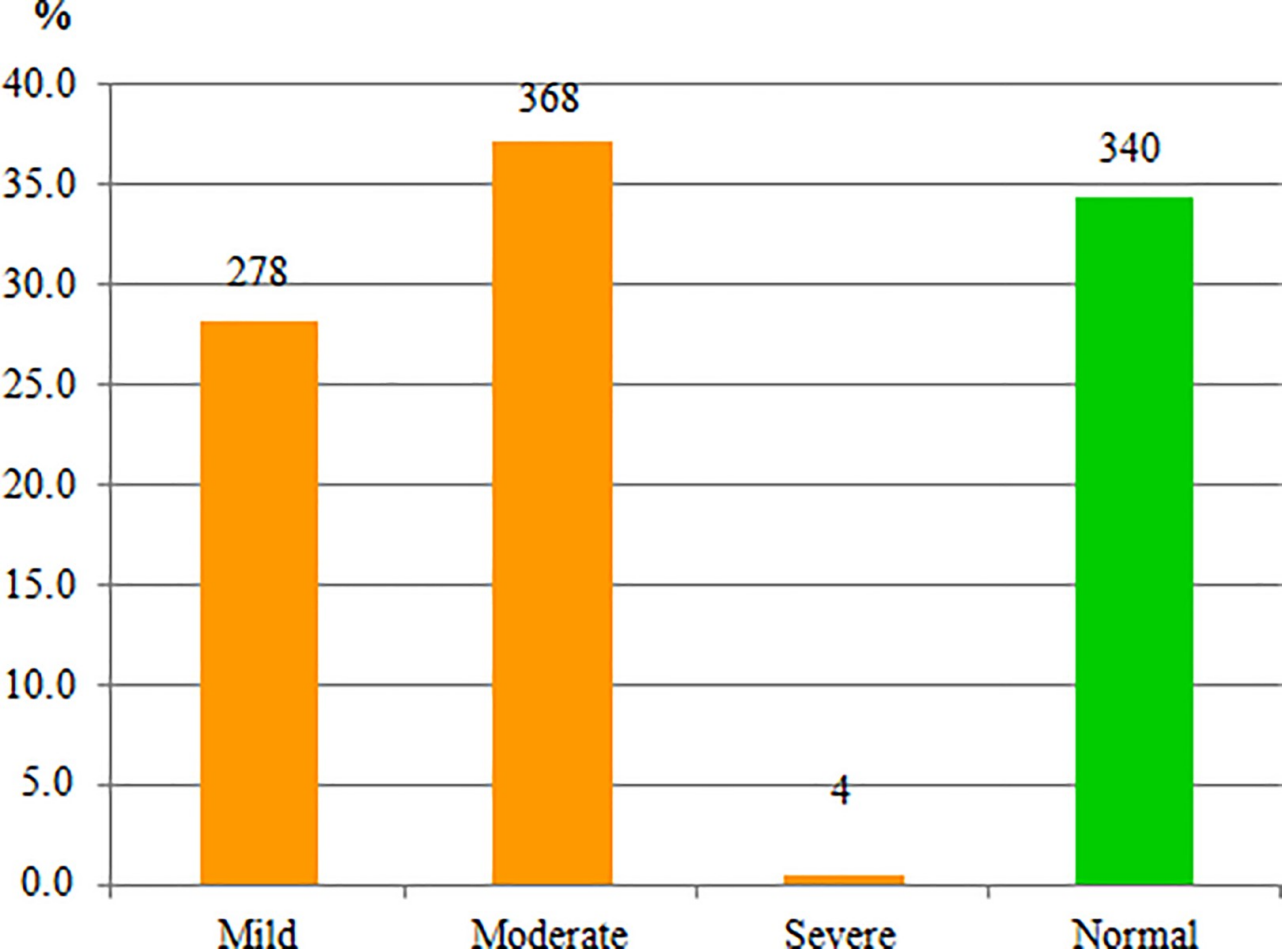
Magnitude and severity of anemia among children aged 6–23 months in rural districts of Wolaita Zone, 2016 (N = 990).

The mean hemoglobin concentration of the children was 10.44 g/dl (95% C.I: 10.36, 10.52 g/dl) with standard deviation of 1.30 g/dl, and with a minimum and maximum hemoglobin concentration of 6.64 to 17.31g/dl. Overall, 650 (65.7%) of the children had anemia with hemoglobin level below 11g/dl (95% C.I: 62.6% - 68.6%). Most of them were mildly (10–10.9 g/dl) and moderately (7–9.9 g/dl) anemic accounting for 278 (28.1%) and 368 (37.2%), respectively; whereas only 4 (0.4%) of children were severely anemic with hemoglobin level below 7 g/dl. The prevalence of anemia decreased as age increased i.e. from as high as 72.6% among infants aged 6–11 months to as low as 61.4% among young children aged 18–23 months “Fig 1”.

### Factors associated with anemia

Bivariate logistic regression showed that age of child, age of mother, religion, occupation of mother, family size, child’s meal frequency, initiation of complementary feeding, receiving anti-helminthic drugs, sex of child and food insecurity were associated with anemia in children (P < 0.05).

However, in the multivariable analysis, age of child, religion, family size and initiation of complementary feeding time were not associated with child anemia (p > 0.05); whereas maternal age and occupation, child’s anti-helminthic drug intake, sex, dietary diversity score and household food insecurity were identified as predictors of child anemia in the multivariable analysis (p < 0.05).

The multivariable logistic regression showed that children of mothers whose age was 35 years and above were two times more likely to be anemic as compared to children of mothers whose age was between 15–24 years, AOR = 1.96 (95% C.I: 1.01, 3.85), p = 0.049. Children of government employee mothers had 79% lower chance of being anemic as compared to children of housewife mothers, AOR = 0.21 (95% C.I: 0.09, 0.47), p<0.001. Similarly, children of merchant mothers had 57% lower chance of getting anemia than children of housewife mothers, AOR = 0.43 (95%C.I: 0.20, 0.92), p = 0.029. In contrary, children of mothers whose occupation was classified as ‘other occupation’ (daily laborers, potters and housemaid) had greater chance of being anemic than children of housewives, AOR = 3.17 (95%C.I: 1.35, 7.43), p = 0.008.

Receiving anti-helminthic drugs was found to be inversely related with childhood anemia. Accordingly, children who received anti-helminthic drugs had 61% lower chance of getting anemia than their counterparts, AOR = 0.39 (95% C.I: 0.24, 0.63), p <0.001. Female children were also twice more likely to be anemic than male children, AOR = 1.76 (95% C.I: 1.30, 2.38), p < 0.001. Nutritional characteristics were also found to be predictive factors of childhood anemia. Accordingly, children who consumed poor dietary diversity (DDS < 4) were found to be more likely anemic than their counterparts, AOR = 1.40 (95% C.I: 1.03, 1.92), p = 0.034. Last but not least, children from moderately food insecure households were two times more likely to be anemic as compared to those from food secure households, AOR = 1.57 (95% C.I: 1.07, 2.32), p = 0.023 (Table 4).

**Table 4:** Multivariable analysis of factors associated with anemia among children in rural districts of Wolaita Zone, 2016 (N = 990).

Variable	Category	Status		COR	95% CI for COR		AOR	95% CI for AOR		P value for AOR
		Anemic	Normal		Lower	Upper		Lower	Upper	
Age of child	6–11 month	244	92							
	12–17 month	182	107	0.64*	0.46	0.90	0.77	0.52	1.12	0.175
	18–23 month	224	141	0.60**	0.44	0.82	0.76	0.52	1.11	0.154
Age of mother	15–24	113	78							
	25–34	447	238	1.30	0.93	1.80	1.52	0.98	2.36	0.061
	35 and above	90	24	2.59**	1.52	4.42	1.96*	1.01	3.85	0.049
Religion	Protestant	571	286							
	Orthodox	67	54	0.62*	0.42	0.91	0.66	0.43	1.02	0.060
	Others^1^	12	0	—	—	—	—	—	—	—
Occupation	Housewife	592	289							
of mother	Government employee	10	26	0.19**	0.09	0.39	0.21**	0.09	0.47	0.000
	Merchant	16	17	0.46*	0.23	0.92	0.43*	0.20	0.92	0.029
	Others^2^	32	8	1.95	0.89	4.29	3.17**	1.35	7.43	0.008
Family size	Below 5	195	112							
	5–7	280	171	0.94	0.70	1.27	0.81	0.55	1.21	0.311
	8 and above	175	57	1.76**	1.21	2.57	1.27	0.79	2.04	0.320
Child’s	<4	394	113							
meal	4–5	241	199	0.35**	0.26	0.46	0.38**	0.27	0.52	0.000
frequency	6–8	15	28	0.15**	0.08	0.30	0.23**	0.10	0.49	0.000
Initiation of	< 6 month	68	22							
complementary	At 6 month	374	240	0.50**	0.30	0.84	0.98	0.55	1.73	0.947
feeding	> 6 month	208	78	0.86	0.50	1.49	0.96	0.53	1.75	0.903
Received anti-helminthic drug	No	609	272							
	Yes	41	68	0.27**	0.18	0.41	0.39**	0.24	0.63	0.000
Sex of child	Male	343	213							
	Female	307	127	1.50**	1.15	1.96	1.76**	1.30	2.38	0.000
Dietary	Below 4	376	192	0.95	0.73	1.23	1.40*	1.03	1.92	0.034
diversity score	4 and above	274	148							
Food insecurity	Food secure	396	232							
	Mildly insecure	48	30	0.94	0.58	1.52	0.93	0.54	1.62	0.809
	Moderately insecure	152	56	1.59**	1.12	2.25	1.57*	1.07	2.32	0.023
	Severely insecure	54	22	1.44	0.85	2.42	1.10	0.62	1.94	0.748

*- P<0.05

**- P<0.01

Others^1^– Catholic, Muslim, Adventist

Others^2^ –Daily laborers, potters, housemaid

## Discussion

Anemia remains one of the major health problems that result in grave health outcomes in developing countries despite the progresses seen in nutrition interventions. The Global Burden of Diseases Study [21] shows that anemia in children is one of the most common causes of child death in Ethiopia, and it continued to be a major public health problem. Similarly, our study showed that two-third of the young children in rural Wolaita had anemia standing as a severe public health problem (above the WHO cut-off point of 40%) [22]. Studies done elsewhere in Ethiopia [23] also reported similar findings although severe anemia was found to be lower in the present study area (0.4%) as compared to Ethiopia’s national rate among young children (3.5%) [12].

UNICEF and WHO recommend adequate breastfeeding, iron supplementation and fortification, and nutrition education for mothers [24] in order to curve health loss (high morbidity and mortality) due to iron deficiency anemia in children. Ethiopia’s nutrition strategy puts weight on the above nutrition intervention with the vision of ensuring adequate micronutrient intake for all children and its citizens [25]. Implementation of the national nutrition strategy with focus to young children will be vital in preventing and treating anemia in rural Wolaita.

The present study showed that maternal age, type of occupation, child’s anti-helminthic drug intake, sex of child, dietary diversity score and household food insecurity were associated with child anemia. Studies have shown that consuming diverse food prevents anemia although this is a difficult option for households in developing countries where there is recurrent food insecurity problem. In conformity to this study, several studies [23, 26, 27] have shown children who had poor dietary diversity score had a higher chance of having anemia. Similarly, WHO report has shown that feeding children diverse foods increases the bioavailability of micronutrients including iron, and this is one of the recommended practices [28]. For instance, eating iron rich food items of animal source such as flesh meat, organ meat, poultry; and non-animal source such as legumes and green leafy vegetables increase the bioavailability of iron and hence enable micronutrient demands for children. Consumption of fruits, vegetables, and tubers that are good sources of vitamins A and C, and folic acid enhances the absorption and utilization of iron [24].

De-worming intestinal parasites also has a positive impact on prevention of anemia among children [24]. Similarly, in this study, children in rural Wolaita who undergone de-worming had lower odds of having iron deficiency anemia. A report of study conducted in 25 Sub-Saharan countries stated that de-worming and iron intake for more than six months prevented anemia in children [15]. De-worming is an essential public health intervention as intestinal parasites, especially hookworm infection, results in intestinal blood loss which in turn contributes to anemia.

Household food security also plays a significant role in preventing anemia among children [24]. In this study, we found that food insecurity was associated with the development of anemia which was higher among children of moderately food insecure households. Studies elsewhere showed similar finding [29], and it is important to address food insecurity problems in rural areas to prevent anemia in children of such areas. In the absence of such measures, it will be difficult to achieve global and national targets of micronutrient problems. The efforts to ensure and assist local people with food security may need implementation of programs funded by government and non-governmental organizations. This could be an important means of intervention if such programs are implemented according to the standard [24] and prioritize people with severe or moderate food insecurity problems. Nevertheless, this study did not attempt to identify households supported by such programs.

Sex was also one of the factors which affected blood hemoglobin level of the children in the present study. Many studies found the correlation between sex and hemoglobin level [29, 30, 31, 32]. According to those studies, the role of sex varied from place to place which gave different gender value for the sex of child with respect to the culture of the community. In the present study, female children were more likely to have anemia than males. Although the exact cause for increased anemia in female children in the present study is difficult to point out, the possible reasons could be high prevalence of sex bias (negatively affecting the feminine gender), late initiation of complementary feeding for female children and the community’s belief to give better care for male children than females [33]. Based on the finding of the present study and other similar studies, female children are not favored to get iron rich food, which increases the risk of iron deficiency anemia in female children. Therefore, it is important to educate the rural community about the importance of providing iron rich food for all children regardless of sex. Further studies may also be required to validate the role of gender in iron deficiency anemia in Ethiopian context.

This study also showed that maternal age and occupation were correlated with hemoglobin level of children. The risk of anemia in children increased with the age of their mothers. In line with this finding, a study in Northeastern Brazil also showed similar trends of iron deficiency anemia [34]. Studies also found that parental occupation was one of the significant predictors of hemoglobin level in children [27]. In this study, mothers who made relatively better income from government employment and trading had a lower chance of having anemic children as compared to housewives. This indicates that anemia is significantly prevalent among families with less educated parents and low income, and hence shows the role of socio-economic inequality in the prevalence of anemia [35]. Therefore, it is important to engage women in income generating activities to make better income and afford supplemental food for their children.

We mention the following limitations of our study. The level of hemoglobin concentration at one point in time was used to diagnose anemia among children. Though this method is the most reliable indicator of anemia, the present study did not aim to identify the etiology of anemia, rather to determine the prevalence and associate anemia with its risk factors. We tried to assess the most common factors of childhood anemia through the dietary assessments and the household food insecurity measurements; however, we did not see the role of genetic factor to cause anemia in the present study for diagnostic limitation within the existing setup [9, 10].

## Conclusion and recommendation

Based on the World Health Organization cut-off point, anemia is found to be a severe public health problem among young children residing in rural districts of Wolaita zone. Nearly two third of children aged 6–23 months were diagnosed to be anemic by having hemoglobin level below 11g/dl. Poor dietary diversity, female sex of child, failure to take anti-helminthic drug, household food insecurity, maternal age and occupation were significantly associated with child anemia.

A large majority of children in the rural Wolaita were anemic, and the need for proven public health interventions such as food diversification, provision of anti-helminthic drugs and ensuring household food security is crucial. In addition, nutrition education and diet diversification through provision of alternative sources of income for women might be useful interventions.

## Supporting information

S1 Questionnaire Questionnaire English version. (DOCX)

S2 Questionnaire Questionnaire Amharic version. (DOCX)

S1 Stata Data Data analyzed by using Stata software. (DTA)

## Abbreviations

ANC: Antenatal Care
AOR: Adjusted Odds Ratio
C.I: Confidence Interval
COR: Crude Odds Ratio
EDHS: Ethiopian Demographic and Health Survey
FANTA: Food and nutrition technical assistant
Hb: Hemoglobin
HFIAS: Household Food Insecurity Access Scale
MUACZ: Mid Upper Arm Circumference Z-score
PCA: Principal Component Analysis
SD: Standard Deviation
SNNPR: Southern Nations, Nationalities and Peoples Region
UNICEF: United Nations Children's Fund
WHO: World Health Organization
