# Supplementary material for: Prevalence and correlates of anemia among children aged 6-23 months in Wolaita Zone, Southern Ethiopia
Source: PLoS One. 2019 Mar 8;14(3):e0206268. doi: 10.1371/journal.pone.0206268 (PMC6407854; doi:10.1371/journal.pone.0206268)
Supplement: S1 Questionnaire — (DOCX) [file pone.0206268.s001.docx]

# Questionnaire

**Prevalence and correlates of anemia among children aged 56-23 months in Wolaita Zone, Southern Ethiopia**

**Section 1.identification**

| **I1** | ID child: \|____\|____\|____\| | **I2** | Date of birth: \|__\|__\|/\|__\|__\|/\|__\|__\| (dd/mm/yy) |
| --- | --- | --- | --- |
| **I3** | District: \|____\| | **I4** | Residence: \|____\| 1. Urban 2. Rural |
| **I5** | Kebele code: \|____\| | **I6** | Id data collector: \|____\|____\| |

**Section 2: socio-demographic and household wealth characteristics**

**2.1. Socio-demographic characteristics of respondents**

| A1 | Current age of mother \|____\|____\| | | | | | | | | |
| --- | --- | --- | --- | --- | --- | --- | --- | --- | --- |
| A2 | Marital status mother: \|___\|  1. Single 2.married/ 3.divorced 4.widowed | | | | | | | | |
| D1 | Ethnicity mother: \|___\|;  1. Wolaita 2.Amhara 3.Gurage 4. Hadiya  5. Kembata 6.others(specify)__________ | | | D2 | Religion mother: \|___\|;  1. Protestant 2. Orthodox 3. Muslim 4.catholic 5. others | | | | |
| D3 | Mother’s occupation \|___\|1. Housewife 2. Farmer 3. Government 4. Merchant 5. Daily laborer 6. NGO 7. Other (specify)__________ | | | | | | | | |
| D4 | Mother’s educational status \|___\| 1. No education 2. Primary 3. Secondary and above | | D5 | | | Husband’s educational status \|___\| 1. No education 2. Primary 3. Secondary and above | | | |
| D6 | Fathers occupation \|___\|1. Farmer 2. Government 3. Merchant 4. Daily laborer 5. NGO 6. Other (specify)__________ | | | | | | | | |
| D7 | Average distance from nearest health center. (minute)______ | | | | | | D8 | How many people live in this house? \|__\|__\| | |
| 2.2. **Household wealth index characteristics of respondents** | | | | | | | | | |
| D9 | Does your household have a functioning …………………..: [write ‘1’ if yes & ‘0’ if no]  D9.1. Television: \|___\| D9.2. Radio: \|___\| D9.3. Satellite dish: \|___\| D9.4. Mobile phone: \|___\| D9.5. Landline phone: \|___\| D9.6. Table: \|___\| D9.7. Chair/bench (not stool): \|___\| D9.8. Bed with sponge mattress:\|__\| D9.9. Bed with straw mattress:\|___\| D9.10. Kerosene lamp: \|___\| D9.11. Pressure lamp\|___\| D9.12. Kerosene stove:\|___\| D9.13. Electric ‘mitad’:\|___\| D9.14. Animal-drawn cart:\|___\| D9.15. Bicycle:\|___\| D9.16. Motorcycle/’bajaj’:\|___\| D9.17. Refrigerator: \|___\| D9.18. Water pump diesel:\|___\| D9a1.1.hoe/mattock \|___\| D9a1.2 hammer/iron bar \|___\| D9a1.3 spade/shovel \|___\| D9a1.4ox plough \|___\| D9a1.5water pump \|___\| D9a1.6tractor \|___\| | | | | | | | | |
| D10 | Animals owned by the household:[for each animal, write the number owned by the household: if the household doesn’t own an animal, write 000]  D10.1. Plow oxen: \|__\|__\|__\| D10.2. Bulls: \|__\|__\|__\| D10.3. Cows/heifers: \|__\|__\|__\| D10.4. Calves: \|__\|__\|__\| D10.5. Sheep & goats: \|__\|__\|__\| D10.6. Horse, donkey & mules: \|__\|__\|__\| D10.7. Chicken: \|__\|__\|__\| D10.8. Bee-hives: \|__\|__\|__\| | | | | | | | | |
| D11 | How many rooms in this household are used for sleeping? \|___\|___\| | | | | | | | | |
| D12 | Housing characteristics | | | | | | | | |
|  | D12.1. Roof: \|___\| | D12.2. Wall: \|___\| | | | | | | | D12.3. Floor: \|___\| |
|  | 1. Thatch/leaf 2. Plastic sheets  3. Corrugated iron sheet 4. Cement  5. Other [specify] ____________ | 1.wooden & mud 2.cartoon 3.cement  4.stone with lime/cement 5.wood plank/shingles  6. Bricks 7. Other[specify]____________ | | | | | | | 1. Earth/ mud 2. Wooden  3. Ceramic tiles 4. Cement/bricks  5. Other [specify]__________ |
| D13 | Type of fuel mainly used for cooking? \|___\|; 1.electricity 2.wood 3.biogas kerosene 4.straw/shrubs/ grass 5.other (specify)____________ | | | | | | | | |
| D14 | Usual place of cooking: \|___\|;  1.in the living house 2.in a separate building 3.outdoors 4.other (specify)________________ | | | | | | | | |
| D15 | Who owns the house you are currently living in? \|___\|  1. Personal 2. Rented from government3. Rented from individuals | | | D16 | | Do you have separate room which is used as kitchen? \|___\|;  0. No 1. Yes | | | |
| D17 | Type of latrine owned by the household: \|___\|;  1.flush /pour flush toilet 2. Ventilated improved pit latrine (vipl) 3. Pit latrine with slab 4. Pit latrine without slab/  5. No facility/bush/field 6. Other (specify) | | | | | | | | |
| D18 | Does any member of this household have a bank account or microfinance saving? \|___\| 0. No 1. Yes | | | | | | | | |

**Section 3: Maternal health status & health service utilization**

I am going to ask you about your health. Please respond to the following questions about you yourself

| **H6** | How many pregnancies have you ever had? *(include current if pregnant)* \|___\| | | **H7** | | How many live born children have you ever had? \|___\| |
| --- | --- | --- | --- | --- | --- |
| **H8** | Have you ever lost a child due to death (including stillbirths)? \|___\| **0**. No **1.** Yes | | **H9** | | If yes, how many? \|___\| children |
| **H10** | Have you ever lost a child due to miscarriage (spontaneous abortion)? \|___\| **0**. No **1.** Yes | | **H11** | | If yes, how many? \|___\| children |
| **H12** | Month interval between the index child and earlier birth? ______months | | **H13** | | In which order is this index child? \|___\| |
| **H14** | Did you receive any antenatal care during the last pregnancy? \|___\|**[if no, skip to h17] 0**. No **1.** Yes | | | | |
| **H15** | How many times did you go for pregnancy checkup? \|___\|  **1.** Once **2**. Twice **3**. Three times **4.**>three times **99**. Don’t remember | | **h16** | | Where did you deliver your last child? \|___\|**1**.home **2**.hospital **3**.health center **4**.health post **5**.other |
| **H20** | Have you encountered any health problems during your last child delivery? \|___\| **0**. No**1**.massive vaginal bleeding  **2**. Retained placenta (> 30 min) **3.birth** canal laceration **4**.unconsciousness **5**. Other (specify) ____ | | | | |
| **K21** | Do you eat additional foods other than common family food \|___\|0. No 1. Yes | | | | |
| **K22** | How many meals do you take per day? \|___\| | **K23** | | How is the amount of food intake compared to previous non-lactating time? \|___\|**1**.increased **2**.decreased **3**.no change | |
| **K24** | Have you ever been sick during the past breast feeding time? \|___\|**0**. No **1**. Yes | **K25** | | If yes for question ____, have you visited health facility? \|___\|**0**. No **1**. Yes | |

**SECTION 4: CHILD HEALTH STATUS**

| **S1.1** | Sex of child \|___\|**1**. Male **2.** Female | S1.2 | Age of child (in months) \|___\|___\| | | | |
| --- | --- | --- | --- | --- | --- | --- |
| **S2** | Has the child ever been immunized?\|___\|**0**. No **1.** Yes | | | | S3 | Does the child have vaccination card? \|___\|**0**. No **1.** Yes |
| **S4** | **I**n the previous six months, did the child take vitamin A? : \|___\| **0**. No **1**. Yes | | | | | |
| **S5** | In the previous six months, did the child take anti-helminthes: \|___\| **0**. No **1**. Yes | | | | | |
|  | **Child illness and symptom** | | | | | |
| **S6** | Has the child had any illness in the past two weeks? **0**. No **1**. Yes  ***If yes, then continue below. Otherwise, skip to the next section.*** | | | | | |
|  | Which illnesses or symptoms has the child had in the past two weeks? ***Do not read the list below;*** | | | For how many days in the past two weeks (14 days), has the Child experienced the symptom? (**00** if child has not experienced the symptom) | | |
|  | **S7** Cough: \|___\| | | | \|___\| \|___\| days | | |
|  | **S8.** Difficult or fast breathing : \|___\| | | | \|___\| \|___\| days | | |
|  | **S9.** fever: \|___\| | | | \|___\| \|___\| days | | |
|  | **S10.** Diarrhea with blood \|___\| | | | \|___\| \|___\| days | | |
|  | **S11.** diarrhea without blood :\|__\| | | | \|___\| \|___\| days | | |
|  | **S12 visible signs of intestinal parasite through mouth/anus** :\|__\| | | | \|___\| \|___\| days | | |

| **S13** | Has the child ever cought malaria ? \|___\|  **0**. No **1**. Yes | | |
| --- | --- | --- | --- |
| **S14** | If yes to the above question, how many times? \|___\| | | |
| **S15** | Has the child caught malaria within the past two weeks? \|___\|  **0**. No **1**. Yes | **S4.10** | Has he received any medical care? \|___\|  **0**. No **1**. Yes |

**SECTION 5: MATERNAL AND CHILD FEEDING INDEX AND DIETARY DIVERSITY**

**First:** Ask the mother to describe the foods (meals and snacks) that her child fed **yesterday during the day and night**, whether at home or outside the home. Start with the first food eaten in the morning & continue probing until she went to sleep. [Write the response under the ‘**CH’** column].

**Then:** Ask the mother to describe all the foods she ate yesterday during the day and night, whether at home or outside the home. [Write the response under the ‘**MO’** column]. [**Write ‘‘1’ if any food from the food group was eaten and ‘0’ if not]**.

| **#** | **FOOD GROUP** | **Child** | **Mother** |
| --- | --- | --- | --- |
| **D1** | CEREALS: Any food made from grains such as maize, millet, wheat, barley, sorghum, rice, teff | \|___\| | \|___\| |
| **D2** | LEGUMES, NUTS & SEEDS: Any food made from legumes such as lentils, beans, guaya, peas, nuts (lewz), sesame(selyit), chickpea | \|___\| | \|___\| |
| **D3** | WHITE TUBERS & ROOTS: Any food made from roots or tubers such as white potatoes, white yams, cassava, false banana (kocho), taro (godere) | \|___\| | \|___\| |
| **D4** | VITAMIN A RICH VEGETABLES & TUBERS:such as pumpkin, carrots, orange/red flushed sweet potatoes, sweet pepper (kariaya) | \|___\| | \|___\| |
| **D5** | DARK GREEN LEAFY VEGETABLES: such as kale (abeshagommen), spinach (kosta), lettuce(selata) | \|___\| | \|___\| |
| **D6** | ANY OTHER VEGETABLES: such as tomato, onion, cabbage (tikilgomen), eggplant, peppers, beets, zucchini, fosoliya, garlic | \|___\| | \|___\| |
| **D7** | VITAMIN A RICH FRUITS: any fruit with yellow or orange flesh such as mango, papaya, cantaloupe, dried apricot, dried peaches | \|___\| | \|___\| |
| **D8** | OTHER FRUITS: all other fruits including wild ones such as avocado, orange, lemon, banana, gishta, | \|___\| | \|___\| |
| **D9** | MILK & MILK PRODUCTS: such as milk, yoghurt, cheese, arera, aguat, or other milk products | \|___\| | \|___\| |
| **D10** | Eggs | \|___\| | \|___\| |
| **D11** | FLESH MEATS: beef, lamb, goat, chicken, wild game or other birds | \|___\| | \|___\| |
| **D12** | ORGAN MEAT: liver, kidney, heart or other organ meats or blood-based foods | \|___\| | \|___\| |
| **D13** | FISH: fresh or dried fish including sardines | \|___\| | \|___\| |
| **D14** | OILS & FATS: oil, fats, butter or ghee added to food or used for cooking | \|___\| | \|___\| |

**SECTION 6 : KAP towards child feeding**

| **C1** | Does the child usually drink tea? \|___\| **0**. No **1**. Yes | **C2** | If yes, when does he drink ? \|___\|  1. Before meal 2. Along with meal 3. After meal |
| --- | --- | --- | --- |
| **C3** | Does the child usually drink coffee? \|___\|**0**. No **1**. Yes | **C4** | If yes, when does he drink ? \|___\|  1. Before meal 2. Along with meal 3. After meal |
| **C5** | Do you bottle-feed your child? **\|___\| 0**. No **1**. Yes | | |
| **C6** | How many times did you give the child complementary feeding in the past 24 hours? \|__\|__\| | | |
| **C7** | How long after birth did you first put (NAME) to the breast? If less than 1 hour, record ‘00’ hours. If less than 24 hours, record hours. Otherwise, record days. **Hours** \|___\|___\| **Days** \|___\|___\| | | |
| **C8** | During the first three days after delivery, did you give (NAME) the liquid that came from your breasts? \|___\|  **0**. No **1.** Yes **98**. Don’t know | | |
| **C9** | In the first three days after delivery, was (NAME) given anything to drink other than breast milk? \|___\|**0**. No **1**. Yes **98**. Don’t know | | |
| **C10** | If yes to the above question, What was (NAME) given to drink? 1. Butter 2. Water 3. Cow milk 4. Porridge 5. Other (specifiy) _______________________ | | |
| **C11** | Are you currently breastfeeding [NAME]?0. No 1. Yes | | |
| **C12** | If no to **K6**, for how many months did you breastfeed (NAME)? If less than one month, record “00” months. Months \|___\|___\| | | |
| **C 13** | If you are currently breast feeding, how many times did you breastfeed [NAME], between sunrise yesterday and sunrise today?  If response is not numeric, probe for a numeric response \|___\| | | |
| **C14** | At what age did you first introduce liquids /foods (semi-solid or solid) other than breast milk to the baby? \|___ \|Months | | |

**Section 7: Household food insecurity access scale (HFIAS)**

| 10.1 | in the past four weeks, did you worry that your household would not have enough food? | (1) yes (2) no 🡪 go to q 10.2 |
| --- | --- | --- |
| 10.1.1 | how many times did this happen in the past month? | \|__\|__\| times |
| 10.2 | in the past four weeks, were you or any household member not able to eat the kinds of foods you preferred because of a lack of resources? | (1) yes (2) no 🡪 go to q 10.3 |
| 10.2.1 | how many times did this happen in the past month? | \|__\|__\| times |
| 10.3 | in the past four weeks, did you or any household member have to eat a limited variety of foods due to a lack of resources? | (1) yes (2) no 🡪 go to q 10.4 |
| 10.3.1 | how many times did this happen in the past month? | \|__\|__\| times |
| 10.4 | in the past four weeks, did you or any household member have to eat some foods that you really did not want to eat because of a lack of resources to obtain other types of food? | (1) yes (2) no 🡪 go to q 10.5 |
| 10.4.1 | how many times did this happen in the past month? | \|__\|__\| times |
| 10.5 | in the past four weeks, did you or any household member have to eat a smaller meal than you felt you needed because there was not enough food? | (1) yes (2) no 🡪 go to q10.6 |
| 10.5.1 | how many times did this happen in the past month? | \|__\|__\| times |
| 10.6 | in the past four weeks, did you or any other household member have to eat fewer meals in a day because there was not enough food? | (1) yes (2) no 🡪 go to q 10.7 |
| 10.6.1 | how many times did this happen in the past month? | \|__\|__\| times |
| 10.7 | in the past four weeks, was there ever no food to eat of any kind in your household because of lack of resources to get food? | (1) yes (2) no 🡪 go to q10.8 |
| 10.7.1 | how many times did this happen in the past month? | \|__\|__\| times |
| 10.8 | in the past four weeks, did you or any household member go to sleep at night hungry because there was not enough food? | (1) yes (2) no 🡪 go to q 10.9 |
| 10.8.1 | how many times did this happen in the past month? | \|__\|__\| times |
| 10.9 | in the past four weeks, did you or any household member go a whole day and night without eating anything because there was not enough food? | (1) yes (2) no 🡪 go to section 11 |
| 10.9.1 | how many times did this happen in the past month? | \|__\|__\| times. |

**Section 8: anthropometry measurement**

|  | **Height/length** | **weight** | **Remark** |
| --- | --- | --- | --- |
|  | **\|__\|__\|. \|__\|** | **\|__\|\|__\|.\|__\|** |  |

**Section 9: Hemoglobin measurement**

**Maternal Hemoglobin**

|  | **Hemoglobin (g/dl)** | **Referral? [0. No 1. Yes]** | **Remark** |
| --- | --- | --- | --- |
| **H1** | **\|__\|__\| .\|__\|** | **\|__\|** |  |

**Child’s Hemoglobin**

|  | **Hemoglobin (g/dl)** | **Referral? [0. No 1. Yes]** | **Remark** |
| --- | --- | --- | --- |
| **H1** | **\|__\|__\| .\|__\|** | **\|__\|** |  |
