# Supplementary material for: Prevalence and correlates of anemia among children aged 6-23 months in Wolaita Zone, Southern Ethiopia
Source: PLoS One. 2019 Mar 8;14(3):e0206268. doi: 10.1371/journal.pone.0206268 (PMC6407854; doi:10.1371/journal.pone.0206268)
Supplement: S2 Questionnaire — (DOCX) [file pone.0206268.s002.docx]

**መጠይቅ**

**በወላይታ ዞን እድሜጣቸዉ 6-23 ወር የሆኑ ህጻናት ላይ የደማነስ በሽታ እና ተያያዥ ችግሮች ምርምር**

**ክፍል 1. መለያ**

| **I1** | መለያ ቁጥር: \|____\|____\|____\| | **I2** | ህጻኑ የተወለደበት ቀን: \|__\|__\|/\|__\|__\|/\|__\|__\| (ቀን/ወር/ዓ.ም) |
| --- | --- | --- | --- |
| **I3** | ወረዳ \|____\| | **I4** | መኖሪያ \|____\| 1. ከተማ 2. ገጠር |
| **I5** | ቀበሌ: \|____\| | **I6** | የመረጃ ሰብሳቢዉ መለያ ቁ.: \|____\|____\| |

**ክፍል2: የስነ-ህዝብ፤ ማህበራዊና የቤተሰብ የሀብት መለኪያ**

**2.1. ስነ-ህዝብና ማህበራዊ ጉዳይ**

| A1 | የእናት እድሜ (ያሁኑ)\|____\|____\| | | A2 | | የእናት እድሜ (ይሄን ህጻን ስትወልድ)\|____\|____\| | |
| --- | --- | --- | --- | --- | --- | --- |
| A3 | የእናት የጋብቻ ሁኔታ: \|___\|  1. ያላገባች 2.ያገባች 3.የተፋታች 4.ባሏ የሞተባት | | A4 | | የእናት ብሄር: \|___\|;  1. ወላይታ 2.አማራ 3.ጉራጌ 4. ሃዲያ  5. ከምባታ 6. .ሌሎች (ይጥቀሱ)____________ | |
| D2 | የእናት ሀይማኖት: \|___\|  1. ፕሮቴሰታንት 2. ኦርቶዶክስ 3. ሙስሊም 4.ካቶሊክ 5.ሌሎች------------ | | | | | |
| D3 | የእናት ትምህርት ደረጃ \|___\|  1. ምንም ያልተማረች 2. የመጀመሪያ ደረጃ (1-8) 3. ሁለተኛ ደረጃ እና ከዚያ በላይ (9 እና ከዛ በላይ) | | | D4 | የባል ትምህርት ደረጃ \|___\|  1. ምንም ያልተማረ 2. የመጀመሪያ ደረጃ (1-8)  3. ሁለተኛ ደረጃ እና ከዚያ በላይ (9 እና ከዛ በላይ) | |
| D5 | የእናት ስራ \|___\|  1. የቤት እመቤት 2 አርሶአደር 3. የመንግስት ስራ 4. ነጋዴ 5. የቀን ስራ 6. ሌሎች (ይጥቀሱ)_____ | | | D6 | የባል ስራ \|___\|  1. አርሶአደር 2. የመንግስት ስራ  3. ነጋዴ 4. የቀን ስራ 5. NGO 6. ሌሎች (ይጥቀሱ)______ | |
| D7 | ከአቅራቢያ ካለዉ ጤና ጣቢያ እና መኖሪያ ቤት መሃል ያለዉ የእግር ጉዞ ርቀት (በደቂቃ) ___________ | | | D8 | እዚህ ቤት ዉስጥ ስንት ሰዉ አለ? \|__\|__\| | |
| 2.2. **የቤተሰብ የሀብት መለኪያ** | | | | | | |
| D9 | ከሚከተሉት ዉስጥ አገልግሎት እየሰጠ ያለ እቃ አለ? …………………..: [ካለ ‘1’ ይጻፉ ከሌለ ‘0’የጻፉ]  D9.1. ተለቪዥን: : \|___\| D9.2. ሬዲዮ:: \|___\| D9.3. ሳተላይት ዲሽ : \|___\| D9.4. ሞባይል ስልክ: \|___\| D9.5. መደበኛ ስልክ: \|___\| D9.6. ጠረጴዛ: \|___\| D9.7. ወንበር (ኩርሲ አያካትትም): \|___\| D9.8. ስፖንጅ ፍራሽ አልጋ :\|___\| D9.9. የገለባ ፍራሽ አልጋ :\|___\| D9.10. ፋኖስ: \|___\| D9.11. ማሾ \|___\| D9.12. ቡታጋዝ:\|___\| D9.13. ኤለክትሪክ ምጣድ:\|___\| D9.14. የፈረስ/ አህያ ጋሪ:\|___\| D9.15. ብስክሌት:\|___\| D9.16. ሞቶር ብስክሌት/’bajaj’:\|___\| D9a1.1. መኮትኮቻ/ዶማ\|___\| D9a1.2 መዶሻ \|___\| D9a1.3 አካፋ \|___\| D9a1.4የእርሻ በሬ \|___\| | | | | | |
| D10 | ቤት ዉስጥ ያሉ እንስሳት ብዛት[የእያንዳዱን እንስሳ ብዛት ቁጥር ይጻፉ፡፡ ቤተሰቡ ምነም እንስሳ ከሌለዉ 000 ይጻፉ]  D10.1. የእርሻ በሬ \|__\|__\|__\| D10.2. ኮርማ (ለእርሻ ያልደረሰ): \|__\|__\|__\| D10.3. ላም \|__\|__\|__\| D10.4. ጥጃ: \|__\|__\|__\| D10.5. በግና ፍየል \|__\|__\|__\| D10.6. ፈረስ፤ አህያ እና በቅሎ \|__\|__\|__\| D10.7. ዶሮ: \|__\|__\|__\| D10.8. የንብ ቀፎ: \|__\|__\|__\| | | | | | |
| D11 | D11.1.የቤቱ ጣሪያ: \|___\| | D11.2. የቤቱ ግድግዳ: \|___\| | | | | D11.3. የቤቱ ወለል: \|___\| |
|  | 1. ሳር/ቅጠል 2. በላስቲክ የተሸፈነ  3. ቆርቆሮ 4. ሲሚንቶ  5. ሌሎች [ይጥቀሱ] ____________ | 1.እንጨትና ጭቃ 2.ክርታስ 3.ሲሚንቶ 4.ድንጋይ በኖራ/ሲሚንቶ 5. ጣዉላ 6. ሸክላ  7. ሌሎች[ይጥቀሱ]___________ | | | | 1. መሬት/ጭቃ/አፈር 2. እንጨት 3. ሴራሚክ 4. ሲሚንቶ/ሸክላ  5.ሌሎች[ይጥቀሱ]______ |

**ክፍል 3.የእናት ጤና ሁኔታና የጤና አገልግሎት አጠቃቀም**

| **H1** | ስንት ግዜ አርግዘሻል? \|___\|  *(እርጉዝ ከሆነች ያሁኑን እርግዝና ጨምሮ)* | | | | | | | | **H2** | ስንት ልጆችን ወልደዋል?  (በህይወት የተወለዱትን ብቻ)? \|___\| | | |
| --- | --- | --- | --- | --- | --- | --- | --- | --- | --- | --- | --- | --- |
| **H3** | እርስዎ ሲወልዱ ሞቶ የተወለደ ልጅ አጋጥሞዎት ያዉቃል? \|___\|  0. አይደለም 1. አዎ 98. አላዉቅም | | | | | | | | **H4** | አዎ ከሆነ ስንት ግዜ? \|___\| | | |
| **H5** | ጽንስ ተጨንግፎብሽ ያዉቃል (በራሱ ግዜ የሚከሰት ዉርጃ)?\|___\| 0. አይደለም 1. አዎ 98. አላዉቅም | | | | | | | | **H6** | አዎ ከሆነ ስንት ግዜ? \|___\| | | |
| **H7** | ይሄ ልጅ ስንተኛ ልጅሽ ነዉ? \|____\| | **H8** | የመጀመሪያዉን ልጅሽን ከወለድሽ በኋላ ምን ያህል ቆይተሸ ነዉ ይህን ልጅ የወለድሽዉ? በወራት ይጻፉ \|___\|___\| (ይሄ የመጀመሪያ ልጅ ከሆነ 00 ዪጻፉ) | | | | | | | | | |
| **H9** | በመጨረሻዉ እርግዝናሽ ጊዜ የጽንስ ክትትል አድርገሽ ታዉቂያለሽ?  (መልሱ አይደለም ከሆነ ወደ ጥያቄ ቁጥር H11 ይሂዱ) \|___\| 0. አይደለም 1. አዎ | | | | | | | | | | **H10** | ተከታትለዉ የሚያዉቁ ከሆነ ለጽንስ ክትትል ስንት ግዜ ሄደዋል?\|___\| |
| **H11** | ይሄን ልጅሽን የት ነዉ የወለድሽዉ? \|___\| 1. ቤት ዉስጥ 2. ጤና ጣቢያ 3.ሆስፒታል 4. ጤና ኬላ 5. ሌላ (ይጥቀሱ) _____ | | | | | | | | | | | |
| **H12** | የመጨረሻዉን ልጅሽን ስትወልጂ የጤና እክል አጋጥሞሻል? \|___\| 0. አይደለም 1. አዎ | | | | | | | | | | | |
| **ከሚከተሉት ዉስጥ ካጋጠማት “1” ይጻፉ ካላጋጠማት “0” ይጻፉ** | | | | | | | | | | | | |
| **H12.1** | ብዙ ደም ፈሶኛል \|___\| | | | **H12.2** | | | እንግዴ ልጅ ቆይቶ ወጣ (ከ 30 ደቂቃ በላይ) \|___\| | | | | | |
| **H12.3** | የማዋለጃ ብልት ቁስለት \|___\| | | | **H12.4** | | | ራስን መሳት \|___\| | | | | | |
| **H12.4** | ሌላ (ይጥቀሱ) _______________________ | | | | | | | | | | | |
| **H13** | ይህን ህጻን ከወለድሽ በኳላ ወባ አሞሽ ያዉቃል? \|___\| 1. አዎ 0. አይደለም | | | | | | | | | | | |
| **H14** | የአልጋ አጎበር ትጠቀሚያለሽ? \|___\| 0. አይደለም 1. አዎ | | | | | **H15** | | ህጻኑ የአልጋ አጎበር ዉስጥ ይተኛል? \|___\| 0. አይደለም 1. አዎ | | | | |
| **M1** | ከቤተሰብ ጋር ከምትመገቢዉ ዉጭ ተጨማሪ ምግብ ትመገቢያለሽ? \|___\| 0. አይደለም 1. አዎ | | | | | | | | | | | |
| **M2** | በቀን ዉስጥ ስንት ግዜ ትመገቢያለሽ? \|___\| | | | | **M3** | | ከመዉለድሽ በፊት ከምትመገቢዉ ጋር ሲነጻጸር ያሁኑ አመጋገብሽ ሁኔታ ምን ይመስላል? \|___\| **1**.ጨምሯል **2**.ቀንሷል **3**.ለዉጥ የለዉም | | | | | |
| **M4** | ይህን ልጅ ከወለድሽ በኋላ አሞሽ ያዉቃል? \|___\|  0. አይደለም 1. አዎ | | | | **M5** | | አሞሽ የሚያዉቅ ከሆነ ለህክምና ወደ ጤና ተቋም ሄደሻል? \|___\|  0. አይደለም 1. አዎ | | | | | |

**ክፍል 4: የህጻኑ ጤና ሁኔታ**

| **S1.1** | የህጻኑ ጾታ \|___\| 1. ወንድ 2. ሴት | **S1.2** | የህጻኑ እድሜ (በወራት ይጻፉ) \|___\|___\| | |
| --- | --- | --- | --- | --- |
| **S2** | ህጻኑ ተከትቧል?\|___\|0. አይደለም 1. አዎ | | **S3** | የክትባት ካርድ አለ*?* \|___\| 0. አይደለም 1. አዎ |
| **S4** | ባለፉት ስድት ወራት ዉስጥ ህጻኑ ቫይታሚን A ወስዷል? : \|___\| 0. አይደለም 1. አዎ | | | |
| **S5** | ባለፉት ስድት ወራት ዉስጥ ህጻኑ የሆድ ዉስጥ ትላትል መድሃኒት ወስዷል?: \|___\| 0. አይደለም 1. አዎ | | | |

|  | **የህጻኑ የህመም ምልክቶች** | | | |
| --- | --- | --- | --- | --- |
| **S6** | ህጻኑ ባለፉት ሁለት ሳምንታት ዉስጥ ታሞ ያዉቃል? \|___\| 0. አይደለም 1. አዎ  ***አዎ ከሆነ ከዚህ በታች ያሉትን ጥያቄዎችን ይመልሱ. ካልሆነ ወደሚቀጥለዉ ክፍል ይሂዱ*** | | | |
|  | **ከዚህ በታች ከተዘረዘሩት የህመም ምልክቶች ዉስጥ ህጻኑ ላይ ከታየ “1” ይጻፉ ካልታየ “0” ይጻፉ፡፡ *ዝርዝሩን አያንብቡ*** | | | |
|  |  | ለስንት ቀን? |  | ለስንት ቀን? |
|  | **S7 .** ሳል: \|___\|  **S8 .** ትኩሳት: \|___\|  **S9.** ተቅማጥ(ደም የሌለበት):\|___\| | \|___\|___\|  \|___\|___\|  \|___\|___\| | **S10.** የአተነፋስ ችግር : \|___\|  **S11.** ተቅማጥ (ደም የተቀላቀለበት) \|___\|  **S12.** ትላትል በአፍ/በፊንጢጣ ታይቶበት ያዉቃል \|___\| | \|___\|___\|  \|___\|___\|  \|___\|___\| |

| **S13** | ከተወለደ ጀምሮ ዎባ አሞት ያዉቃል ? \|___\|  0. አይደለም 1. አዎ | | |
| --- | --- | --- | --- |
| **S14** | አዎ ከሆነ ስልት ግዜ? \|___\| | | |
| **S15** | ባለፉት ሁለት ሳምንታት በዎባ ታሟል ? \|___\|  0. አይደለም 1. አዎ | **S4.10** | ከታመመ ህክምና ተሰጥቶታል? \|___\|  0. አይደለም 1. አዎ |

**ክፍል 5: የህጻኑና የእናት አመጋገብ ሁኔታ**

መጀመሪያ ህጻኑ ትናንትና ቀንና ማታ የተመገባቸዉን የምግብ አይነቶችን ይጠይቁ፡፡ ጥያቄዎትን ከቁርስ ይጀምሩ፤ ከዛም ሌሎችንም ምግቦችን መጠየቅ ይቀጥሉ፡፡ መልሱን **‘CH’** ስር ይሙሉ፡፡

ቀጥሎ እናቲዬዋ የተመገበቻቸዉን የምግብ አይነቶች ይጠየቁ መልሱን **‘MO’** ስር ይሙሉ].

ከተዘረዘሩ የምግብ አይነቶች ዉስጥ የተመገቡት **ካለ ‘‘1’ ይጻፉ፡፡** ከዝርዝሮቹ ዉስጥ ምንም **ካልበሉ ‘0’ ይጻፉ.**

| **#** | **የምግብ አይነት** | **ህጻን** | **እናት** |
| --- | --- | --- | --- |
| **D1** | በቆሎ፤ ማሽላ፤ ስንዴ፤ ገብስ፤ ሩዝ፤ ጤፍ | \|___\| | \|___\| |
| **D2** | ምስር፤ አተር፤ ባቄላ፤ ጉዋያ፤ ለዉዝ፤ ሰሊጥ፤ ሽምብራ | \|___\| | \|___\| |
| **D3** | ስራስር (ድንች፤ቦይና፤ ቦዬ፤ እንሰት፤ ቆጮ) | \|___\| | \|___\| |
| **D4** | ካሮት፤ ብርቱካን፤ ዱባ፤ ስኳር ድንች፤ ቃሪያ | \|___\| | \|___\| |
| **D5** | አበሻ ጎመን፤ ቆስጣ፤ ሰላጣ | \|___\| | \|___\| |
| **D6** | ቲማቲም፤ ጥቅል ጎመን፤ ፎሶሊያ፤ ዝንጅብል | \|___\| | \|___\| |
| **D7** | ማንጎ፤ ፓፓያ፤ ኮክ | \|___\| | \|___\| |
| **D8** | ሌሎች ፍራፍሬዎች (አቮካዶ፤ ሎሚ፤ ሙዝ፤ ጊሽጣ) | \|___\| | \|___\| |
| **D9** | የወተት ዉጤቶች (እርጎ፤ አይብ፤ አሬራ) | \|___\| | \|___\| |
| **D10** | እንቁላል | \|___\| | \|___\| |
| **D11** | ስጋ (የበሬ፤ የጠቦት፤ ፍየል፤ በግ፤ ዶሮ ...) | \|___\| | \|___\| |
| **D12** | ስጋ (ጉበት፤ ኩላሊት፤ ልብ፤ ጣፊያ ...) | \|___\| | \|___\| |
| **D13** | አሳ | \|___\| | \|___\| |
| **D14** | ዘይት፤ ጮማ፤ ቅቤ | \|___\| | \|___\| |

**ክፍል 6 : የህጻኑ አመጋገብ ሁኔታ**

| **C1** | ህጻኑ ሻይ ይጠጣል? \|___\|  0. አይደለም 1. አዎ | **C2** | አዎ ከሆነ መቼ መቼ ነዉ የሚጠጣዉ ? \|___\|  1. ከምግብ በፊት 2. ከምግብ ጋር 3. ከምግብ በኋላ |
| --- | --- | --- | --- |
| **C3** | ህጻኑ ቡና ይጠጣል? \|___\|  0. አይደለም 1. አዎ | **C4** | አዎ ከሆነ መቼ መቼ ነዉ የሚጠጣዉ ? \|___\|  1. ከምግብ በፊት 2. ከምግብ ጋር 3. ከምግብ በኋላ |
| **C5** | ህጻኑ በጡጦ ይጠባል? **\|___\|** 0. አይደለም 1. አዎ | | |
| **C6** | ባለፉት 24 ሰዓታት ዉስጥ ህጻኑ ስንት ግዜ ነዉ ምግብ የተመገበዉ ? [የሚጠጣ ነገር አያካትትም!] \|__\|__\| | | |
| **C7** | ህጻኑ ከተወለደ በኋላ ከምን ያህል ሰዓት በኋላ ነዉ ጡት ያጠባሽዉ? ከአንድ ሰዓት በታች ከኖነ ‘00’ ይጻፉ፡፡ ከ 24 ሰዓት በታች ከሆነ ሰዓት ይጻፉ፡፡ ከ 24 ሰዓት በላይ ከሆነ ቀን ይጻፉ፡፡ **ሰዓት** \|___\|___\| **ቀን** \|___\|___\| | | |
| **C8** | ከተወለደ በኋላ እንገር (የመጀመሪያዉን የጡት ወተት) ለህጻኑ ሰጥተሻል? \|___\| 0. አይደለም 1. አዎ | | |
| **C9** | ህጻኑ እንደተወለደ ከጡት ወተት ዉጪ ሌላ ነገር ሰተሸዋል? \|___\| 0. አይደለም 1. አዎ | | |
| **C10** | አዎ ከሆነ ምን ተሰጠዉ? ______________________________________________ | | |
| **C11** | ህጻኑ አሁን ጡት ይጠባል? **\|___\|** 0. አይደለም 1. አዎ | | |
| **C12** | አሁን የማይጠባ ከሆነ ለስንት ወር ነዉ ያጠባሽዉ? ከአንድ ወር በታች ከሆነ “00” ይጻፉ፡፡ \|___\|___\| | | |
| **C13** | አሁን የሚጠባ ከሆነ በ 24 ሰዓት ዉስጥ ስንት ግዜ ታጠቢያለሽ? \|___\| | | |
| **C14** | ለህጻኑ ተጨማሪ ምግብ የጀመርሺዉ በስንተኛዉ ወሩ ነዉ? \|___ \| | | |

**ክፍል 7 : የቤተሰብ የምግብ ዋስትና መለኪያ**

| 10.1 | ባለፉት አራት ሳምንታት ዉስጥ ቤተሰብዎ በቂ ምግብ ላይኖረዉ ይችላል ብለዉ ሰግተዉ ያዉቃሉ? | (1) አዎ  (2) አይደለም 🡪 ከሆነ ወደ ጠያቄ ቁ.10.2 ይሂዱ |
| --- | --- | --- |
| 10.1.1 | ባሳለፉት ወር ዉስጥ ስንት ግዜ ነዉ ይህ የሆነዉ? | \|__\|__\| ግዜ |
| 10.2 | ባለፉት አራት ሳምንታት ዉስጥ እርስዎም ሆነ ሌላ የቤተሰብ አባል ከአቅም (ከገንዘብ) ማነስ የተነሳ ለመብላት የተመኙትን የምግብ አይነት ሳይበሉ የቀሩበት ቀን አለ? | 1) አዎ  (2) አይደለም 🡪 ከሆነ ወደ ጠያቄ ቁ.q 10.3ይሂዱ |
| 10.2.1 | ባሳለፉት ወር ዉስጥ ስንት ግዜ ነዉ ይህ የሆነዉ? | \|__\|__\| ግዜ |
| 10.3 | ባለፉት አራት ሳምንታት ዉስጥ እርስዎም ሆነ ሌላ የቤተሰብ አባል ከአቅም (ከገንዘብ) ማነስ የተነሳ መብላት ካለበት የምግብ አይነቶች የተወሰኑትን (ጥቂቶችን) ብቻ የበሉበት ሁኔታ አለ? | (1) አዎ  (2) አይደለም 🡪 ከሆነ ወደ ጠያቄ ቁ.q 10.4ይሂዱ |
| 10.3.1 | ባሳለፉት ወር ዉስጥ ስንት ግዜ ነዉ ይህ የሆነዉ? | \|__\|__\| ግዜ |
| 10.4 | ባለፉት አራት ሳምንታት ዉስጥ እርስዎም ሆነ ሌላ የቤተሰብ አባል ከአቅም (ከገንዘብ) ማነስ የተነሳ ለመብላት የማይፈልጉትን ምግብ በልተዉ ያዉቃሉ? | (1) አዎ  (2) አይደለም 🡪 ከሆነ ወደ ጠያቄ ቁ.q 10.5ይሂዱ |
| 10.4.1 | ባሳለፉት ወር ዉስጥ ስንት ግዜ ነዉ ይህ የሆነዉ? | \|__\|__\| ግዜ |
| 10.5 | ባለፉት አራት ሳምንታት ዉስጥ እርስዎም ሆነ ሌላ የቤተሰብ አባል ከአቅም (ከገንዘብ) ማነስ የተነሳ ከሚያስፈልግዎ ምግብ ያነሰ በልተዉ ያዉቃሉ? | (1) አዎ  (2) አይደለም 🡪 ከሆነ ወደ ጠያቄ ቁ.q10.6ይሂዱ |
| 10.5.1 | ባሳለፉት ወር ዉስጥ ስንት ግዜ ነዉ ይህ የሆነዉ? | \|__\|__\| ግዜ |
| 10.6 | ባለፉት አራት ሳምንታት ዉስጥ እርስዎም ሆነ ሌላ የቤተሰብ አባል ከአቅም (ከገንዘብ) ማነስ የተነሳ በቀን ዉስጥ ከሚመገበዉ ያነሰ ቁጥር ተመግበዉ ያዉቃሉ? (ምሳሌ፡ ቁርስ፤ ምሳ፤ እራት ….) | (1) አዎ  (2) አይደለም 🡪 ከሆነ ወደ ጠያቄ ቁ.q 10.7ይሂዱ |
| 10.6.1 | ባሳለፉት ወር ዉስጥ ስንት ግዜ ነዉ ይህ የሆነዉ? | \|__\|__\| ግዜ |
| 10.7 | ባለፉት አራት ሳምንታት ዉስጥ እርስዎም ሆነ ሌላ የቤተሰብ አባል ከአቅም (ከገንዘብ) ማነስ የተነሳ በቤት ዉስጥ ምግብ ጠፍቶ ያዉቃል? | (1) አዎ  (2) አይደለም 🡪 ከሆነ ወደ ጠያቄ ቁ.q10.8ይሂዱ |
| 10.7.1 | ባሳለፉት ወር ዉስጥ ስንት ግዜ ነዉ ይህ የሆነዉ? | \|__\|__\| ግዜ |
| 10.8 | ባለፉት አራት ሳምንታት ዉስጥ እርስዎም ሆነ ሌላ የቤተሰብ አባል ከአቅም (ከገንዘብ) ማነስ የተነሳ እየራበዉ በቂ ምግብ ሰላልነበረ ማታ ተኝቶ (አንቀላፍቶ) ያዉቃል? | (1) አዎ  (2) አይደለም 🡪 ከሆነ ወደ ጠያቄ ቁ.q 10.9ይሂዱ |
| 10.8.1 | ባሳለፉት ወር ዉስጥ ስንት ግዜ ነዉ ይህ የሆነዉ? | \|__\|__\| ግዜ |
| 10.9 | ባለፉት አራት ሳምንታት ዉስጥ እርስዎም ሆነ ሌላ የቤተሰብ አባል ከአቅም (ከገንዘብ) ማነስ የተነሳ ምንም ምግብ ሳይበላ ሙሉ ቀንና ማታ ያሳለፈ ሰዉ አለ? | (1) አዎ  (2) አይደለም 🡪 ከሆነ ወደ ክፍል 10 ይሂዱ |
| 10.9.1 | ባሳለፉት ወር ዉስጥ ስንት ግዜ ነዉ ይህ የሆነዉ? | \|__\|__\| ግዜ. |

**ክፍል 8: የህጻን ቁመትና ክብደት**

|  | **ቁመት/ርዝመት** | **ክብደት** | **ምርመራ** |
| --- | --- | --- | --- |
|  | **\|__\|__\|. \|__\|** | **\|__\|\|__\|.\|__\|** |  |

**ክፍል 9: Hemoglobin**

**የእናት Hemoglobin**

|  | **Hemoglobin (g/dl)** | **ረፈራል [0. አይደለም 1.አዎን]** | **ምርመራ** |
| --- | --- | --- | --- |
| **H1** | **\|__\|__\| .\|__\|** | **\|__\|** |  |

**የልጅ Hemoglobin**

|  | **Hemoglobin (g/dl)** | **ረፈራል [0. አይደለም 1.አዎን]** | **ምርመራ** |
| --- | --- | --- | --- |
| **H1** | **\|__\|__\| .\|__\|** | **\|__\|** |  |
